# CDCA4 as a novel molecular biomarker of poor prognosis in patients with lung adenocarcinoma

**DOI:** 10.3389/fonc.2022.865756

**Published:** 2022-09-15

**Authors:** Jianlong Tan, Fengyu Chen, Bin Ouyang, Xiuying Li, Weidong Zhang, Xinglin Gao

**Affiliations:** ^1^ The Second School of Clinical Medicine, Southern Medical University, Guangzhou, China; ^2^ Department of Geriatric Respiratory Medicine, Guangdong Provincial People’s Hospital, Guangdong Academy of Medical Sciences, Guangdong Provincial Geriatrics Institute, Guangzhou, China; ^3^ Department of Pulmonary and Critical Care Medicine, Hunan Provincial People’s Hospital, The First Affiliated Hospital of Hunan Normal University, Changsha, China

**Keywords:** CDCA4, lung adenocarcinoma, biomarker, prognosis, TCGA

## Abstract

**Background:**

Because of the high incidence and poor prognoses of lung adenocarcinoma (LUAD), it is essential to identify cost-effective treatment options and accurate and reliable prognostic biomarkers. CDCA4 upregulation has been identified in many cancers. However, the prognostic importance of CDCA4 and its role in LUAD remain unknown.

**Methods:**

CDCA4 expression was assessed through IHC, Western blotting (WB) and RT-PCR. The Cancer Genome Atlas (TCGA) provided data from 513 patients to study the expression and prognostic relevance of CDCA4 in LUAD. This study used gene set enrichment analyses (GSEA), gene ontology and KEGG pathway analyses for elucidating potential mechanisms underpinning the function of CDCA4 in LUAD. We also investigated correlations between immune infiltration and CDCA4 expression with single specimen GSEA (ssGSEA).

**Results:**

According to database analysis and identification of patient tissue samples, CDCA4 expression in tumour tissues surpassed that in normal tissues (P< 0.001). Increased CDCA4 expression was positively correlated with a higher T, N, pathologic stage and poor primary therapy outcome. In addition, the Kaplan–Meier plotter exhibited that an elevated CDCA4 expression was related to worse disease-specific survival(DSS) and overall survival (OS) (DSS HR= 5.145, 95% CI=3.413-7.758, P<0.001; OS HR=3.570, 95% CI=2.472-5.155, P<0.001). Then multivariate COX regression analyses indicated that the CDCA4 gene was an independent risk consideration for prognoses. GO and KEGG results showed that CDCA4 and its neighbouring genes were enriched in the cell cycle and DNA replication. As determined by GSEA, CDCA4 was related to various immune-related signalling pathways (SPs), Homologous recombination, DNA replication and the cell cycle. SsGSEA analysis showed a significant association between CDCA4 expression and Th2 cells, mast cells, eosinophils and Th17 cells.

**Conclusions:**

CDCA4 expression is increased in LUAD and is a potential predictive biomarker and therapeutic target.

## Introduction

Lung cancer is the most common type of cancer and the primary reason for cancer deaths globally (1). Unfortunately, most lung cancer patients are not detected until the metastatic stage (2). Despite continued advances in treatment strategies (like immunotherapy, targeted therapy, chemotherapy, radiotherapy and surgery), the prognoses remain bleak, with a five-year relative survival rate of only 19.7% in China (3). The five-year relapse-free survival (RFS) after surgical resection is only 70% (4). Based on histological type, non-small cell lung cancer (NSCLC) made up approximately 85% of lung cancer, while lung adenocarcinomas (LUAD) made up nearly 40% (5). LUAD possesses a high burden of tumour mutations such as EGFR, HER2, BRAF, ROS1, ALK, and KRAS (6–9). The discovery of these biomarkers has revolutionized the therapeutic landscape of advanced LUAD. However, the prognosis of LUAD remains unsatisfactory because of its remarkable heterogeneity and aggressiveness. Therefore, developing novel tumour biomarkers with high specificity and sensitivity is crucial for the early detection, treatment and prognosis of LUAD.

The cell division cycle-associated (CDCA) protein family (CDCA1-8) is involved in the cell cycle, which is closely related to carcinogenesis (10, 11). Efforts have been made to identify CDCA genes as biomarkers for the development and prognosis of different malignancies (12, 13). The up-regulation of CDCA gene expression may play a vital role in ovarian cancer oncogenesis through the PLK1 pathway (13). CDCA4, known as HEPP/TRIP-Br3/SEI-3 as well, is associated with the G1/S transition transcription factor. It encodes a protein member of the E2F family of transcription factors involved cell cycle regulation and DNA synthesis (14). According to earlier studies, through the E2F/retinoblastoma protein pathway, CDCA4 controls cell proliferation and E2F-dependent transcriptional activation (15). Various studies have extensively confirmed the close relationship between CDCA4 upregulation and tumorigenesis (12, 13, 16). There is evidence that overexpression of CDCA4 stimulates proliferation and inhibits apoptosis in MCF-7/ADM human breast cancer cells (16). CDCA4 regulates the mRNA expression of the JUN oncogene and acts as a critical determinant of cell fate (17). Notably, overexpression of CDCA4 is directly associated with reduced post-progression survival (PPS) in ovarian cancer (13). CDCA4 has been validated as a prognostic biomarker for various malignancies (12, 18). Wu et al. and colleagues found that increased CDCA4 mRNA expression was strongly related to survival in patients featuring squamous cell carcinoma of the head and neck (12). However, the link between CDCA4 expression and LUAD remains to be fully explored.

The objectives of this study were 1) to understand whether CDCA4 expression correlates with clinical and pathological aspects in patients with LUAD; 2) to investigate the predictive value of CDCA4 in LUAD; 3) to evaluate the expression model of CDCA4 in tumour and peritumor lung tissues, and 4) to understand the underlying mechanisms using bioinformatics analysis. In addition, an online tumour infiltration immune cell tool was employed to assess the association between CDCA4 expression and the clinical characteristics of LUAD.

## Materials and methods

### Clinical samples

From January to December 2020, 39 patients with LUAD underwent surgery in the Department of Thoracic Surgery at Hunan Provincial People’s Hospital and paired tumour and normal tissue (>5 cm proximity) specimens adjacent to the tumour were collected for real-time quantitative PCR (RT-qPCR), WB, and immunohistochemical (IHC) experiments. The clinical characteristics of the 39 individuals can be obtained in **Supplementary Tables 1**, **2**. All selected patients received surgery without neoadjuvant therapy, autoimmune diseases or other malignant tumors. The ethics committee of Hunan Provincial People’s Hospital authorized this study (No.202049). All patients completed written informed consent, and no additional special treatments were administered preoperatively.

### Immunohistochemistry

LUAD tissues which contained 39 tumours and paired normal tissues were used for immunohistochemical staining of CDCA4. Hematoxylin and eosin staining of the tumour and normal tissues close to the tumour followed by immunohistochemical staining. The main staining procedure was as follows. Four-millimetre-thick paraffin sections were dewaxed with xylene and washed in an ethanol gradient. After antigen retrieval featuring EDTA buffer (pH=9.0), endogenous peroxidase activity was eliminated by adopting 3% H_2_O_2_ for 10 minutes and incubated with CDCA4 polyclonal antibody (1:300; No. YT0820; ImmunoWay Biotechnology, USA) at 4°C overnight. After rinsing three times with PBS, add the primary antibody and incubate them with the secondary antibody (Goat Anti-Rabbit IgG Antibody-HRP; No.201105S407q; Maixin Biotech Co., Ltd, Fuzhou, China) for 50 min at room temperature(RT). After rising in PBS for three minutes, incubate the sections and stain them with a DAB colour development kit (Fuzhou Maixin Biotech Co., Ltd., China), followed by hematoxylin staining, drying and mounting. CDCA4 expression was quantified in at least five locations under 200x magnification based on staining intensity (0–3) and the proportion of positively stained tumour cells (0-100%). The following are the scoring guidelines. 0 denotes no staining; 1 denotes weak yellow-brown staining; 2 denotes modest yellow-brown staining; and 3 denotes severe yellow-brown staining (strong staining, brown). The latter were classified as follows: 0 (negative); 1 (approximately 25% positive cells); 2 (approximately 25% to 50% positive cells); 3 (approximately 51 to 75% positive cells); and 4 (approximately >75% positive cells). Immunostaining was reviewed separately by two professional pathologists who kept the clinical results of the patients confidential.

### Quantitative real-time PCR analysis

Quantify CDCA4 expression by employing real-time reverse transcription-polymerase chain reaction (RTPCR). Isolate total RNA from tumours, and adjacent normal tissues of 10 patients by applying Trizol reagent (TianGen, Beijing, China) and reverse transcribe them into cDNA using RevertAid reverse transcriptase (Thermo, USA) according to the producer’s instructions. Perform qRT-PCR by employing PerfectStart Green qPCR 2x SuperMix (TransGen Biotech, China) and the Q1 Real-Time System (ABI), with GAPDH as an internal reference. Use the primers below in the qRT-PCR. CDCA4: 5’-CACGAGGACTGAAGAGGAAATGT-3’ (forward); 5’-TTGGGCTCCACAAGCATGTG-3’ (reverse); GAPDH: 5’-CCAGGTGGTCCTGA-3’ (forward); 5’- CCAGGTGCTCCTGA-3’ (reverse). Calculate all mRNA levels by utilizing the 2Ct approach, and test all samples in triplicate using this method.

### Western blotting

Based on the producer’s instructions, isolate total proteins from tissues of 10 patients using the RIPA protein extraction reagent (Beyotime, China). Afterwards, measure protein contents by adopting a BCA kit (Beyotime, China). Proteins were separated and transferred to PVDF membranes using 10% SDS-PAGE (Merck Millipore, Germany). Block the films by employing 5% skimmed dry milk in PBST (1 PBS + 0.1% Tween-20) buffer for 2 h at RT. After rinsing the membranes by utilizing PBST, incubate them by employing anti-CDCA4 (1:5,000, Proteintech, USA) primary antibody at 4°C overnight before incubating them by utilizing horseradish peroxidase-conjugated secondary antibody (1:5,000) for one hour at RT. Wash blots three times with PBST, and quantify protein levels with a ClinxChemiScope 6000 (Clinx Scientific Instruments, China) before visualization using enhanced chemiluminescence. Internal controls were identified as GAPDH, and relative expression was normalized to GAPDH. Perform the experiment in triplicate.

### Data collection

In May 2020, the RNA-seq gene data and associated clinicopathological characteristics were downloaded from the TCGA database (https://portal.gdc.cancer.gov ) as Level 3 gene expression data. The following analysis stage was to convert the level 3 HTSeq-FPKM data into transcripts per million reads (TPM). LUAD patients lacking sufficient survival and/or expression data were excluded. R software (version 3.6.2) was applied for analyzing the data of each RNA-Seq gene expression level 3 and the clinical information of LUAD patients (19). Finally, data from 513 patients, including 57 paired LUAD tissue and para-cancerous tissue samples, were downloaded. Among the enrolled patients, according to the American Joint Committee on Cancer (AJCC) 8th edition staging manual for lung cancer, this study included 274 patients (53.41%) at stage I, 121 patients (23.59%) at stage II, 84 patients (16.37%) at stage III, and 10 patients (1.95%) at stage IV (**Table 1**).

**Table 1:** The clinicopathological characteristics of LUAD in patients with TCGA.

Characteristics	N% or median (range)
Age	
>65	262 (51.07%)
≤65	241 (46.98%)
Data missing	10 (1.95%)
Gender (%)	
Female	276 (53.80%)
Male	237 (46.20%)
path T-stage (%)	
T1	168 (32.75%)
T2	276 (53.80%)
T3	47 (9.16%)
T4	19 (3.70%)
Data missing	3 (0.59%)
path N-stage (%)	
N0	330 (64.33%)
N1	95 (18.52%)
N2	74 (14.42%)
N3	2 (0.39%)
Data missing	12 (2.34%)
path M-stage (%)	
M0	344 (67.06%)
M1	25 (4.87%)
Data missing	144 (28.07%)
Pathologic stage (%)	
Stage I	274 (53.41%)
Stage II	121 (23.59%)
Stage III	84 (16.37%)
Stage IV	10 (1.95%)
Data missing	24 (4.68%)
Primary therapy outcome (%)	
CR	315 (61.40%)
PD	68 (13.26%)
PR	6 (1.17%)
SD	37 (7.21%)
Data missing	87 (16.96%)
TP53 status (%)	
Mut	241 (46.98%)
WT	267 (52.05%)
Data missing	5 (0.97%)

### CDCA4 correlation genes analysis

After removing the repeated sequences of patients’ tumours, we used the Pearson method of cor. test function in R (version: 4.0.2) to detect the TPM expression and CDCA4-related genes in 513 tumours. *P* < 0.05 and |R| > 0.2 were statistically, and finally we got 6952 significant correlation genes of CDCA4.

### Gene-set enrichment analyses

Gene Ontology (GO) and Kyoto Encyclopedia of Genes and Genomes (KEGG) pathway enrichment analyses were conducted by employing the R package clusterProfiler (version: v3.18.1) for correlation genes with CDCA4. For identifying over-represented GO terms in three categories (cellular component, molecular function and biological courses), and the KEGG pathway, the R package enrichplot(version: v1.10.2) was adopted to visualize. For these analyses, *p* < 0.05 and *q* < 0.2 were regarded to denote statistical significance.

### Gene set enrichment analysis

To further determine the function of genes related to CDCA4, we sequenced these genes in LUAD tumours in the TCGA data set according to the relationship between these genes and CDCA4. Then “gseGO” and “gseKEGG” function of the R package clusterProfiler (version: v3.18.1) was used to analyze GO_ BP and KEGG. We set a statistically significant p-value of 0.05 for GO_BP and KEGG enrichment analyses.

### Immune infiltration analysis by single sample gene set enrichment analysis

We assessed the infiltration of 24 immune cell types (ICTs) in tumour tissues by employing the ssGSEA approach of the Gene Set Variation Analysis (GSVA) package (http://www.bioconductor.org/packages/relaease/bioc/html/GSVA.html) of R software (version 3.6.2). The ssGSEA scored the absolute expression of genes in each tumour sample and calculated an enrichment score according to the marker genes of the 24 ICTs found in the literature (20). The Spearman correlation and Wilcoxon rank-sum tests were adopted for assessing the association between the immune cells and CDCA4 and the relationship between CDCA4 low and high expression groups and immune cell infiltration.

### Statistical analysis

R and IBM SPSS 26.0 were applied for evaluating and performing a statistical study of the data. To investigate the association between CDCA4 and clinicopathological characteristics, Pearson χ^2^ tests and univariate logistic regression were utilized. The cor.test package in R was used to calculate Pearson correlations, and the ggpubr and corrplot packages in R were used to create correlation graphs. The association between clinical variables and DSS or OS time in patients with TCGA-LUAD was investigated by employing the Kaplan-Meier (KM) technique and COX regression analysis. Multivariate Cox analyses were utilized to determine the effect of CDCA4 expression combined with other clinicopathological variables in survival. The median expression level was used to define the cut-off point of CDCA4 expression. Each hypothesis test was two-sided, and statistical significance was defined as a *P* of 0.05. In addition, a prognostic nomogram model was created based on multivariate regression results to provide an accurate multivariate clinical prognostic evaluation method for patients. A nomogram was created by applying the rms R package (http://cran.r-project.org/web/packages/rms/index.html). Afterwards, a calibration plot was created to test its predictive power.

## Results

### Elevated expression of CDCA4 in LUAD

**Figure 1:**
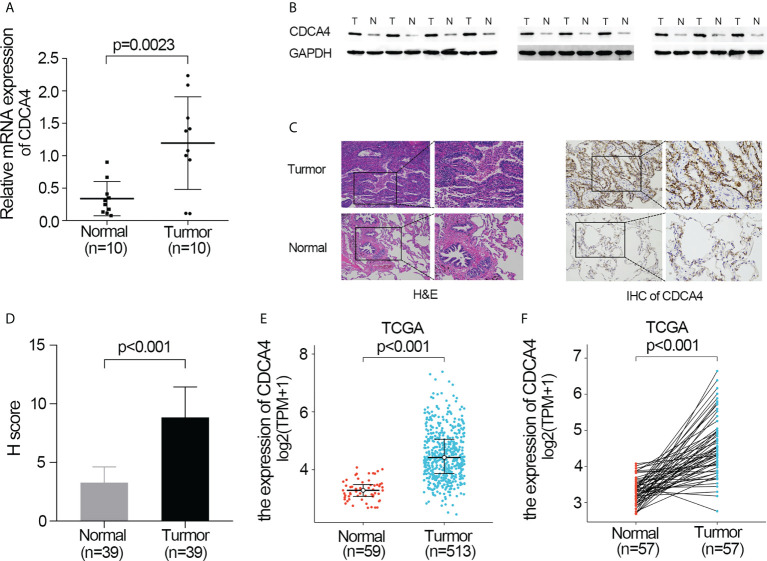
Validate the expression of CDCA4 in LUAD. **(A)** qRT-PCR was used to identify the expression of CDCA4 mRNA in tumors and adjacent tissues of 10 LUAD patients. **(B)** Western blotting was used to determine the expression of CDCA4 protein in tumors and adjacent tissues of 10 LUAD patients. **(C)** CDCA4 expression in LUAD tissues and adjacent normal tissues assayed by IHC (×200) and (×400). **(D)** H score of IHC staining of LUAD tissues and adjacent tissues. **(E)** CDCA4 mRNA levels in LUAD tissues from the TCGA database. **(F)** CDCA4 mRNA expression levels in the tumors and adjacent tissues of 57 LUAD patients from the TCGA database. Data are expressed as mean standard deviation (SD). Normal, lung tissue; tumor, lung adenocarcinoma tissue; TCGA, The Cancer Genome Atlas.

First, qRT-PCR and WB analyses were employed for evaluating CDCA4 expression in clinical LUAD samples. In comparison to normal human lung tissue, CDCA4 expression in LUAD was elevated (**Figures 1A, B**). Next, immunohistochemical analysis revealed that CDCA4 was mainly located in the cytoplasm and film of tumour cells and exhibited little expression in the normal lung cells. In LUAD, the average scores were 8.85 ± 2.89, whereas the para tumour samples score was 3.28 ± 1.32 (p<0.001; **Figures 1C, D**). This conclusion was further verified using the TCGA datasets. It was identified that CDCA4 expression levels in 513 tumour tissues substantially surpassed those in normal tissues based on TCGA data (*P*<0.001; **Figure 1E**). CDCA4 expression was then analyzed using matched plots between LUAD adjacent and tumour samples from the same individuals. CDCA4 expression in 57 tumour tissues surpassed that in 57 matched adjacent tissues (*P*<0.001; **Figure 1F**). Collectively, the results prove that CDCA4 mRNA and protein contents were significantly upregulated in LUAD tissues.

### CDCA4 upregulation was associated with unfavourable clinicopathological features

**Figure 2:**
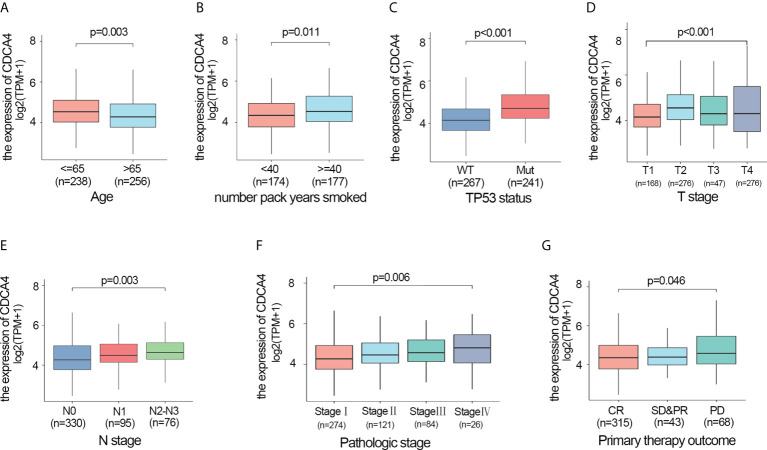
Higher CDCA4 expression was associated with unfavourable clinicopathological characteristics in LUAD. **(A–G)** CDCA4 expression was strongly correlated with younger age, more years of smoking, patients’ T-stage, N-stage, pathological stage, poor primary therapy outcome and more P53 mutation (*P*<0.05).

We also investigated whether there was an association between CDCA4 expression and clinical and pathological features. According to the Kruskal-Wallis rank-sum test and Wilcoxon rank-sum test, higher CDCA4 levels were associated with younger age (*P*=0.003), more years of smoking (*P*=0.011), T stage, N stage, advanced pathologic stage, poor primary therapy outcome and more P53 mutation (**Figures 2A–G**, *P*<0.05). As exhibited in **Table 2**, univariate logistic regression analyses of CDCA4 expression exhibited that high CDCA4 was obviously related to unfavorable characteristics such as younger age [OR=0.61 (0.43-0.87) for >65 vs. <=65, *P*=0.007], more years of smoking [OR=1.53 (1.00-2.33) for >=40 vs. 40<, *P*=0.049], larger primary tumour extent in LUAD [OR=2.21 (1.52-3.25) for T2-4 vs. T1, *P <*0.001], more severe regional lymph node invasion [OR=1.98 (1.36-2.90) for N1-3 vs N0, *P*<0.001], poor primary therapy result [OR=1.89 (1.12-3.25) for PD vs. SD-CR, *P*=0.045], higher incidence of P53 mutations [OR=0.31 (0.21-0.44), *P*< 0. 001], and higher pathologic stage [OR=1.80 (1.26-2.57) for stage II-IV vs. stage I, *P=0*.001]. Together, these results suggest CDCA4 upregulation was related to unfavourable clinicopathological features in LUAD patients.

**Table 2:** Relationship between CDCA4 expression and clinicopathological characteristics of TCGA database by logistic regression analysis.

Characteristics	Total number (N)	Odds Ratio (OR)	*P* value
Age (>65 vs. <=65)	494	0.61 (0.43-0.87)	0.007
Smoker (Yes vs. No)	499	1.46 (0.89-2.42)	0.137
number pack years smoked (>=40 vs. <40)	351	1.53 (1.00-2.33)	0.049
T stage (T2-4 vs. T1)	510	2.21 (1.52-3.25)	<0.001
N stage (N1-3 vs. N0)	501	1.98 (1.36-2.90)	<0.001
M stage (M1 vs. M0)	369	2.00 (0.87-5.03)	0.116
Primary therapy outcome (PD vs. SD-CR)	426	1.89 (1.12-3.25)	0.019
TP53 status (Mut vs. WT)	508	3.28 (2.28-4.73)	<0.001
Pathologic stage (Stage II- IV vs. Stage I)	505	1.80 (1.26-2.57)	0.001

### Survival outcomes and multivariate examination

**Figure 3:**
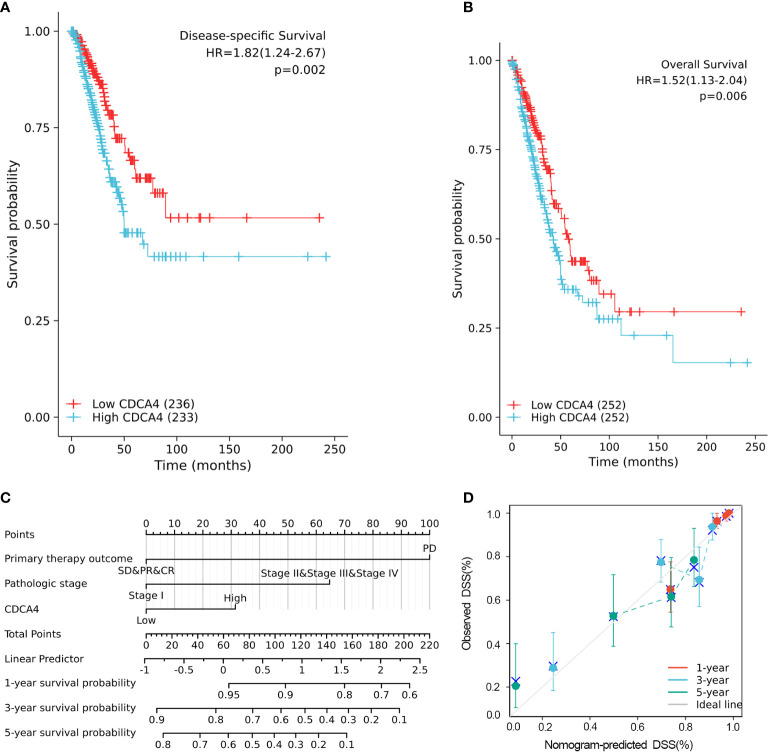
Higher CDCA4 expression was associated with poorer prognosis in LUAD. **(A, B)** KM survival analyses exhibited that patients with high CDCA4 levels had worse prognoses of DSS and OS (DSS HR=1.82,95% CI=1.24-2.67, *P*=0.002; OS HR=1.52; 95% CI=1.13-2.04, *P*=0.006). **(C)** Nomogram constructed using DSS related clinical factors and CDCA4. **(D)** Calibration plots showing good agreement with the best performance for DSS. DSS, disease-specific survival.

The disease-specific survival (DSS) and OS of the two CDCA4 expression value groups were assessed to determine their predictive relevance. According to **Figures 3A, B**, KM survival analyses exhibited that patients with high CDCA4 levels had worse prognoses of DSS and OS (DSS HR=1.82,95% CI=1.24-2.67, *P*=0.002; OS HR=1.52; 95% CI=1.13-2.04, *P*=0.006). In addition, univariate and multivariate analyses were conducted by employing the Cox proportional hazards model (CPHM). According to univariate analyses (**Table 3A**; **Supplementary Table 3A**), high CDCA4 levels and T stage, N stage, M stage, pathologic stage and primary therapy outcome were related to low DSS and OS. Finally, multivariate analysis showed that high CDCA4 levels (DSS HR=1.674; 95% CI=1.112-2.521, P=0.014; OS HR=1.427, 95% CI=1.017-2.003, P=0.04), advanced pathologic stage (DSS HR=2.885, 95% CI=1.868-4.456, P<0.001; OS HR=2.462, 95% CI=1.731-3.501, P<0.001) and poor primary therapy outcome (DSS HR= 5.145, 95% CI=3.413-7.758, P<0.001; OS HR=3.570, 95% CI=2.472-5.155, P<0.001) were independently related to poor prognosis (**Table 3B**; **Supplementary** **Table 3B**). This data implies that CDCA4 may be a useful biomarker for predicting LUAD.

**Table 3:** Univariate and multivariate analysis of the relationship between disease specific survival and clinicopathologic characteristics in patients with TCGA.

Characteristics	Total number (N)	HR (95% CI)	*P* value
A			
Age (>65 vs. <=65)	459	1.039 (0.713-1.513)	0.842
Gender (Female vs. Male)	469	1.046 (0.720-1.519)	0.815
Smoker (Yes vs. No)	455	1.013 (0.585-1.755)	0.962
number pack years smoked (>=40 vs. <40)	318	0.904 (0.569-1.437)	0.67
T stage (T2-4 vs. T1)	466	1.747 (1.125-2.714)	0.013
N stage (N1-3 vs. N0)	457	2.795 (1.919-4.071)	<0.001
M stage (M1 vs. M0)	327	2.480 (1.278-4.811)	0.007
Pathologic stage (Stage II-IV vs. Stage I)	461	3.519 (2.350-5.271)	<0.001
Primary therapy outcome (PD vs. SD-CR)	408	5.929 (3.981-8.830)	<0.001
TP53 status (Mut vs. WT)	465	1.335 (0.920-1.937)	0.128
CDCA4 (High vs. Low)	469	1.823 (1.243-2.675)	0.002
B			
Pathologic stage (Stage II-IV vs. Stage I)	461	2.885(1.868-4.456)	<0.001
Primary therapy outcome (PD vs. SD-CR)	408	5.145(3.413-7.758)	<0.001
CDCA4 (High vs. Low)	469	1.674(1.112-2.521)	0.014

### Risk score model for nomogram

A nomogram consisting of independent prognostic variables was then constructed to quantify the risk assessment and probability of survival for individual LUAD patients. A score was assigned to each variable based on a multivariate Cox proportional hazards model. The multivariate Cox proportional hazards model was adopted for scoring each variable. The weighted scores calculated using all variables were used to estimate the predicted DSS and OS at 1-, 3-, and 5- years. The calculated c-index for predicted DSS and OS was 0.782(95% CI=0.757-0.806), 0.717 (95% CI=0.692-0.742), respectively, indicating that the nomogram was a good predictor for DSS and OS (**Figure 3C**; **Supplementary Figure 1A**). In the calibration survey, the 1-, 3-, and 5-year prediction lines for the estimated likelihood of survival showed high agreement with the ideal performance (45-degree dashed line) (**Figure 3D**; **Supplementary Figure 1B**).

### Identification of CDCA4 co-expressed differential genes

**Figure 4:**
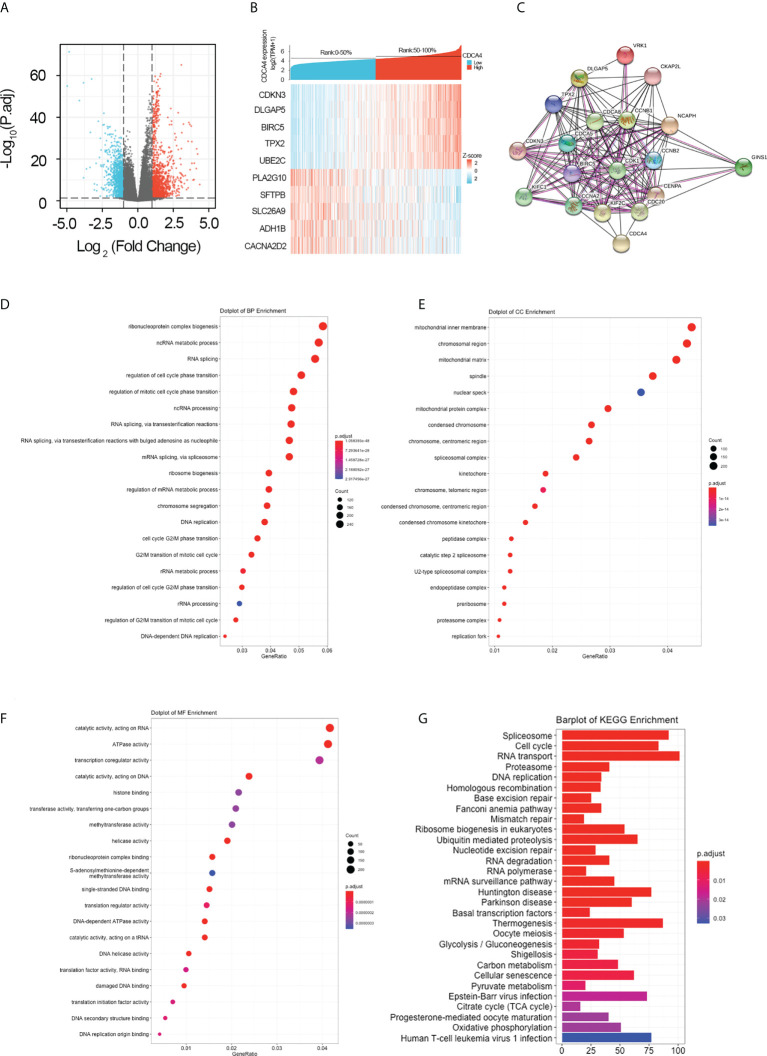
Genes co-expressed by CDCA4 and biological functions of CDCA4 associated with LUAD. **(A)** A volcano plot of differential gene profiles between high and low CDCA4 groups shows that 179 genes were up-regulated and 77 were down-regulated (adjusted *P*-value of 0.01 and |log2-fold change [FC]| > 2). **(B)** A heat map illustrating the positive co-expression of ten representative CDCA4 genes in LUAD. Data was normalized using the Z-score normalization method. **(C)** PPI network created and displayed using CDCA4 co-expressed genes from http://string-db.org. **(D)** BP outcomes; **(E)** CC outcomes; **(F)** MF outcomes from Metascape analysis of functionally enriched GO. **(G)**. KEGG results based on the expression levels of CDCA4 in the LUAD and TCGA datasets. PPI, protein-protein interaction; KEGG, Kyoto Encyclopedia of Genes and Genomes; BP, biological process; CC, cellular component; MF, molecular function.

For further elucidating the biological function of CDCA4 in LUAD, we downloaded the co-DEGs profile of CDCA4 from the TCGA public database. 256 DEGs, comprising 179 upregulated genes and 77 downregulated ones, were significantly associated with CDCA4 expression according to the criteria of *P*<0.05 and |logFC|>2. The overall closely co-expressed genes of CDCA4 in LUAD were shown as a volcano plot map (**Figure 4A**). Subsequently, these aberrant genes were shown as a heat map (**Figure 4B**). The PPI network of these 256 common genes was then constructed by STRING based on the correlation coefficients to understand the underlying mechanisms better. The top 20 genes were selected and visualized for analysis (**Figure 4C**).

### GO and KEGG enrichment analysis identified pathways modulated by CDCA4 in LUAD

**Figure 5:**
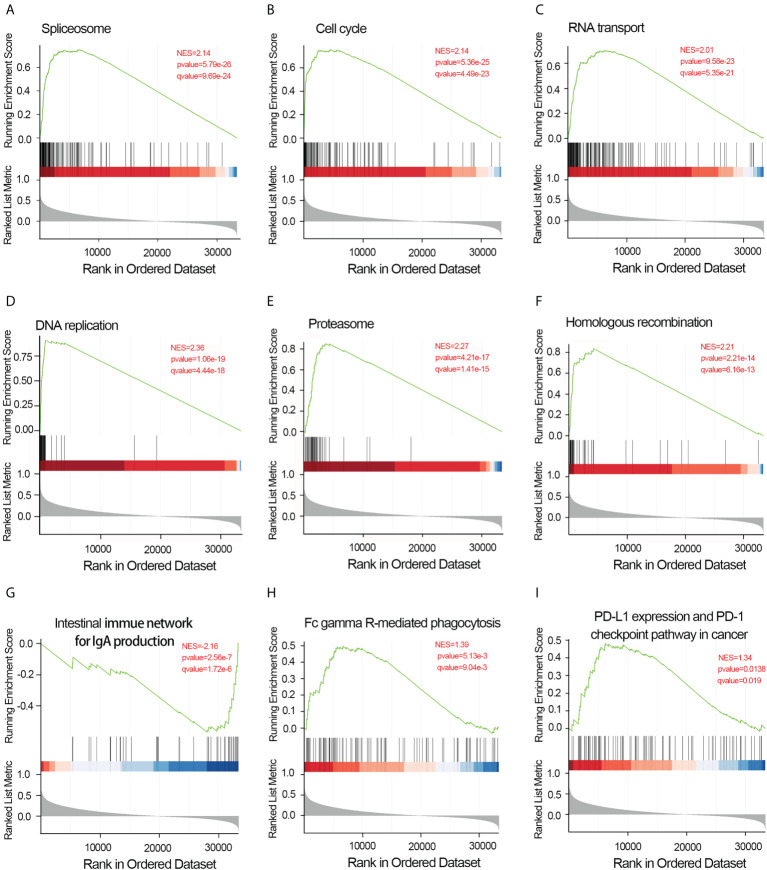
Results of gene set enrichment analysis of CDCA4. **(A)** Spliceosome. **(B)** Cell cycle. **(C)** RNA transport. **(D)** DNA replication. **(E)** Proteasome. **(F)** Homologous recombination. **(G)** Intestinal immune network for IgA generation. **(H)** Fc gamma R-mediated phagocytosis.**(I)** PD-L1 expression and PD-L1 checkpoint pathway in cancer. NES, normalized enrichment score; FDR, false discovery rate.

To elucidate the potential function of CDCA4 in LUAD progression, we performed GO annotation and KEGG pathway analyses. Various BPs, CCs and MFs. CDCA4 and its adjacent genes were significantly enriched in the biogenesis of ribonucleoprotein complex, the regulation of G2/M phase transition of the cell cycle, G2/M phase transition of mitotic cell cycle, G2/M phase transition of the cell cycle, DNA replication, ncRNA metabolic process and RNA splicing (**Figure 4D**). The molecular functions of these genes include single-stranded DNA binding, acting on DNA, ATPase activity, acting on RNA and catalytic activity (**Figure 4E**). The cellular components of these genes comprise kinetochore, condensed chromosome, centromeric region, chromosome, chromosomal region and spliceosomal complex. (**Figure 4F**). KEGG path analyses exhibited that CDCA4 was related to genes involved in the Base excision repair, Homologous recombination, DNA replication, Proteasome, RNA transport, Cell cycle and Spliceosome. (**Figure 4G**). Furthermore, GSEA indicated that in the high or low CDCA4 expression group, PD-L1 expression and PD-L1 checkpoint pathway in cancer, Fc gamma R-mediated phagocytosis, intestinal immune network for IgA generation, homologous recombination, proteasome, DNA replication, RNA transport, cell cycle and Spliceosome were enriched (**Figures 5A–I**). The outcomes imply that CDCA4 upregulation may influence LUAD progression *via* Cell cycle, homologous recombination and DNA replication.

### The relationship between immune cell infiltration and CDCA4 expression in lung adenocarcinoma

For determining the association between CDCA4 expression and immune cell infiltration (ICI) in the LUAD microenvironment, we first used ssGSEA with the Wilcoxon rank-sum test for assessing the difference among 24 different immune cell types of LUAD patients based on CDCA4 expression. It presented an apparent rise in immunological infiltration and heterogeneity. The proportion of follicular helper T cells (Tfh cells), T central memory (TCM), T cells, NK CD56 (bright) NK cells, plasmacytoids (pDCs), NK cells, Mast cells, immature DCs (iDCs), Eosinophils, CD8 T cells and B cells was significantly high in the CDCA4 low-expression group, and the proportion of activated DCs (aDCs), Th2, T gamma delta (Tgd) and CD56 (dim) NK cells was significantly high in the CDCA4 high-expression group (**Supplementary Figure 2**).

**Figure 6:**
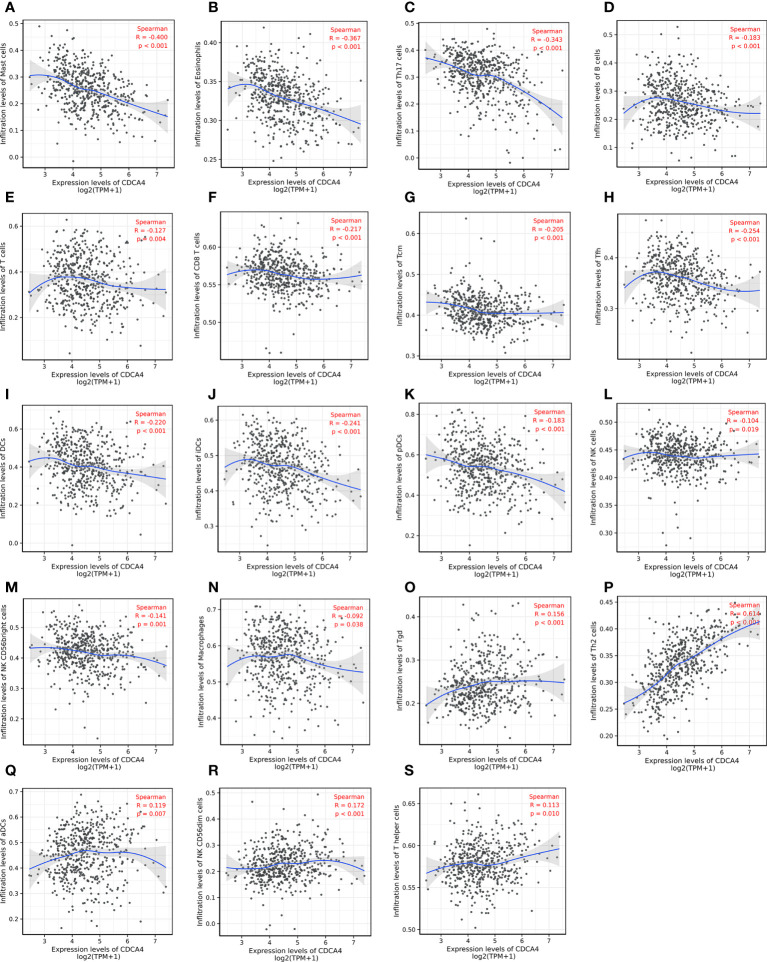
Investigation of the correlation between CDCA4 expression and immune cell infiltration in LUAD(P<0.05). **(A-N)** CDCA4 expression was negatively correlated with the infiltration of Mast cells, Eosinophils, Th17, B cells, T cells, CD8 T cells, T central memory, follicular helper T cells, DCs, immature DCs, pDCs, NK cells, NK CD56 (bright) cells, and Macrophages. **(O-S)** CDCA4 expression was positively correlated with T gamma delta, Th2, activated DCs, NK CD56 (dim) cells, T helper cells.Data were assessed by Spearman correlation analysis.

Then we used Spearman correlation analysis for determining the association between CDCA4 expression and ICI in the LUAD microenvironment. **Figures 6A–N** exhibited that CDCA4 expression had a negatively correlation with the infiltration of Mast cells, Eosinophils, Th17, B cells, T cells, CD8 T cells, T central memory, follicular helper T cells, DCs, immature DCs, pDCs, NK cells, NK CD56 (bright) cells, and Macrophages. Also, a significantly positive correlation was found with T gamma delta, Th2, activated DCs, NK CD56 (dim) cells, T helper cells (**Figures 6O–S**). As indicated by these findings, CDCA4 could be key to regulating ICI in the tumour microenvironment (TME).

## Discussion

Lung cancer is the most typical fatal disease in China and a severe global public health problem (21). Despite significant advances in recent years, the incidence and mortality of lung cancer continue to rise. Investigation of prognostic factors is a critical component of precision medicine and will value treatment allocation (22). CDCA4, encoding 241 amino acids, is on chromosome 14. *In vitro* studies on breast, cervical and malignant melanoma have investigated the expression and function of CDCA4 in tumorigenesis (16, 17, 23). Multiple signalling pathways, especially classical signalling pathways involved in carcinogenesis, are closely associated with CDCA4, and several investigations have shown that CDCA4 over-expression is also related to poor prognoses in various malignancies (17, 23, 24). However, the effect of CDCA4 on LUAD remains a mystery to us. Therefore, it is necessary to further understand the role of CDCA4 in LUAD and its prognostic significance, as well as the regulatory mechanism supporting its role.

CDCA4 is aberrantly regulated in various cancers, comprising triple-negative breast cancer (BC), Wilm’s tumour, melanoma, and osteosarcoma, and is associated with poor patient outcomes (13, 25–28). Shaul et al. found that CDCA4 is highly expressed in BC tissue in comparison to normal tissues using the MERAV database (29). Using the ONCOMINE database, Chen et al. compared CDCA4 gene transcriptional data between standard samples and tumour tissues, resulting in a 2.213-fold change in CDCA4 (13). However, there is a little study to explore CDCA4 expression in lung adenocarcinoma. In this investigation, we used qRT-PCR, Western blotting and IHC on paired LUAD and standard lung tissue samples and found increased expression of CDCA4 in LUAD tissues (**Figures 1A–D**, *P*<0.001). This result was consistent with datasets from TCGA (**Figures 1E, F**).

Furthermore, ovarian cancer patients with elevated CDCA4 expression levels were related to the lower post-progression survival (13). CDCA4 was also upregulated in neck and head squamous cell carcinoma tissues; nonetheless, the higher expression of CDCA4 was associated with more prolonged relapse-free survival (12). In addition, Ran et al. reported elevated CDCA4 expression in patients with squamous cell carcinoma or lung adenocarcinoma; however, no further studies were performed (30). In the current investigation, increased CDCA4 expression was associated with unfavorable clinicopathological characteristics and worse prognoses (**Figures 2**, **3**). In univariate and multivariate analysis, increased CDCA4 expression was confirmed as an independent adverse prognostic factor (**Table 3**, **Supplementary Table 3**). In addition, nomograms combining CDCA4 expression and other independent prognostic variables showed a better prediction of DSS and OS in patients with LUAD (**Figure 3**, **Supplementary Figure 3**). These results may help in the development of an effective biomarker.

Aberrancy in cell cycle progression is an essential mechanism underpinning tumorigenesis (31, 32). It is reported that CDCA4 can be transferred to the centrosome during mitosis and then to the intermediate region. Interference with CDCA4 RNA may damage spindle function during chromosome segregation, or lead to abnormal cell division, resulting in multinucleate and multipolar spindles (33). In addition, CDCA4 may act as a “traffic cop”, affecting mRNA expression of Jun proto-oncogenes and directing upstream signals to the protective elements to determine cell fate (17). In this study, GO analysis exhibits that CDCA4 is involved in courses highly related to tumorigeneses, like DNA replication, modulation of G2/M transition of mitotic cell cycle, modulation of cell cycle phase transition, modulation of G2/M transition of the cell cycle, modulation of mitotic cell cycle phase transition and DNA-dependent DNA replication (**Figures 4D–F**). Then, KEGG analyses exhibited genes co-expressed with CDCA4 in the spliceosome, DNA replication, cell cycle, proteasome and RNA transport (**Figure 4G**). We further validated these results by using GSEA, which indicated that CDCA4 overexpression was collected with Spliceosome, Fc gamma R-mediated phagocytosis, IgA production by the intestinal immune network, homologous recombination, proteasome, DNA replication, RNA transport, cell cycle, PD-L1 expression and PD-1 checkpoint pathway (**Figure 5**). CDCA4 is associated with the destiny of BC cells, and downregulation of CDCA4 in human BC cells *in vitro* may inhibit proliferation while promoting apoptosis (16). Down-regulation of CDCA4, a miR-15a target, leads to cell cycle arrest in malignant melanoma cells in the G0/G1 phase (23). CDCA4 silencing impeded the transition from S to G2, leading to a reduction in cell growth and proliferation of triple-negative BC cells *in vivo* and *in vitro* (26).

Moreover, interference of CDCA4 significantly increased the fraction of the G0/G1 phase of MCF−7/ADM human BC cells and reduced its proliferation by inducing apoptosis (16). Ran et al. reported that over-expression of miR-15a-5p of A549 cells raised the ratio of the G1 phase, and inhibited cell proliferation, clonal formation, and invasion *in vitro*. Furthermore, they reported that CDCA4 constituted a candidate target for miR-15a-5p. The outcomes indicate that CDCA4 is closely related to tumour progression in LUAD by influencing the cell cycle (30). In lung squamous cell carcinoma (LUSC), CDCA4 overexpression significantly inhibited apoptosis, and enhanced the invasion and migration *in vitro*, leading to a deterioration of LUSC progression (34). However, Xu et al. reported that inhibiting CDCA4 induced Epithelial-Mesenchymal Transition, invasion and migration of NSCLC cells while suppressing autophagy of NSCLC cells (35). The inconsistent results of numerous studies suggest that CDCA4 can be involved in a more complicated regulatory network, and its specific regulatory mechanisms remain unknown.

In addition, CDCA4 is related to immune infiltrates in lung adenocarcinoma. The outcome of the connection between CDCA4 and TIICs indicated CDCA4 might play a role in modulating ICI (**Figure 6**). CDCA4 showed the closest relationship with Th2 cells (**Figure 6P**, **Supplementary Figure 2C**). The group with high CDCA4 expression had more Th2 cells but lower mast cells, eosinophils and Th17 cells (**Supplementary Figure 2**). Moreover, the GSEA analyses exhibited CDCA4 affects various immune-associated signalling pathways (**Figures 5G–I** **)**. The quantity, type, and location of immune cells in the tumour microenvironment (TME) influence disease development and progression (36). There are a wide variety of immune cells like macrophages, B and T lymphocytes, and mast cells that can infiltrate tumours, and their composition and organization within the TME are closely linked to cancer patients’ clinical outcomes (37, 38). T cells constitute adequate immune cells. Miller et al. first discovered elevated Th2-type responses in basal cell carcinomas, whereas benign tumours showed a predominance of Th1-type responses, implying dominant expression of Th2-type factors in malignant tumours (39). Thereafter, in a range of malignancies, including lung and cervical cancers, a significant predominance of Th2-type cytokines and Th1/Th2 imbalance was found in cancer tissues and immune cells from patients’ peripheral blood (40–42). The interaction between T lymphocytes and NSCLC cells within the TME is essential to NSCLC development (43). As the tissue-resident, innate immune cell, Mast cells contribute to the cancer microenvironment by modulating various tumour biology events. Salamon et al. found that the internalization of tumour-derived microvesicles from NSCLC cell lines can enhance mast cell migratory capability and increase TNF-α and MCP-1 release, thereby affecting tumorigenesis (44). Th17 cells feature complicated biological functions in cancer development. Ye et al. reported that high counts of pleural Th17 cells in malignant pleural effusion are related to promoted survival of NSCLC (45). However, little study focused on the relationship between CDCA4 and tumour-infiltrating immune cells. Only one previous study determined that CDCA4 regulates monocyte adhesion, leukocyte infiltration, and cytotoxicity of tumour cells (46). Therefore, plans for further understanding the CDCA4-medicated crosstalk with TIICs in the TME are necessary, which may help understand tumour progression and develop probable therapeutic modalities.

However, there are certain limitations. Firstly, there are inconsistent treatments and a lack of clinical information in public databases as the experiments were conducted in various laboratories. Secondly, potential molecular mechanisms of CDCA4 in carcinogenesis have not been investigated. We have formulated several plans for further wet lab work soon to explore the relevant signalling pathways of CDCA4 in LUAD.

## Conclusion

The present outcomes exhibit that CDCA4 levels are significantly higher in LUAD samples and are linked with unfavourable clinicopathological characteristics and poor prognoses of LUAD patients. Furthermore, CDCA4 is related to immune infiltrates in LUAD. In addition, CDCA4 may promote the progression of LUAD by regulating spliceosome, Fc gamma R-mediated phagocytosis, intestinal immune network for IgA production, homologous recombination, proteasome, DNA replication, RNA transport, Cell cycle, PD-L1 expression and PD-1 checkpoint pathway, making it an attractive prognostic biomarker for LUAD. Nonetheless, additional experimental investigations are required to determine the underlying processes and therapeutic effects in patients with LUAD.

## Data availability statement

The original contributions presented in the study are included in the article/**Supplementary Material**. Further inquiries can be directed to the corresponding author.

## Ethics statement

The studies involving human participants were reviewed and approved by Hunan Provincial People’s Hospital. The patients/participants provided their written informed consent to participate in this study.

## Author contributions

TJL and GXL designed and analyzed this study. TJL, CFY, LXY, OYB and ZWD collected the data. TJL and GXL wrote and revised the manuscript. All authors contributed to and approved the final version of the manuscript.

## Funding

This study was supported by Hunan Provincial Health Commission (Grant Nos.202203023386), the RENSHU funding of Hunan Provincial People’s Hospital (Grant Nos.201911) and the Science and Technology Planning Project of Guangdong Province (2017A070701014).

## Acknowledgments

The authors gratefully acknowledge the contribution of the TCGA database and the Molecular Signatures Database.

## Conflict of interest

The authors declare that the research was conducted in the absence of any commercial or financial relationships that could be construed as a potential conflict of interest.

## Publisher’s note

All claims expressed in this article are solely those of the authors and do not necessarily represent those of their affiliated organizations, or those of the publisher, the editors and the reviewers. Any product that may be evaluated in this article, or claim that may be made by its manufacturer, is not guaranteed or endorsed by the publisher.
