# Supplementary material for: CDCA4 as a novel molecular biomarker of poor prognosis in patients with lung adenocarcinoma
Source: Front Oncol. 2022 Sep 15;12:865756. doi: 10.3389/fonc.2022.865756 (PMC9520321; doi:10.3389/fonc.2022.865756)
Supplement: Supplementary file 7 [file DataSheet_7.docx]

library(IOBR)

library(EPIC)

library(estimate)

library(tidyverse)

library(tidyHeatmap)

library(maftools)

library(ggpubr)

library(ggplot2)

library(survival)

library(biomaRt)

library(UCSCXenaTools)

library(dplyr)

library(clusterProfiler)

library(msigdbr)

library(org.Hs.eg.db)

library(GDCRNATools)

library(enrichplot)

library(ReactomePA) #gsePathway

library(grid) #grobTree

library(data.table) #

##################

###TPM表达值-分析#

##################

luad_tpm<-read.table(file="/Users/zhaoguofeng/work-Geoff/personal/Tan_Hunan_Clinical_hospital_LUAD_ceRNA/TCGA-LUAD/LUAD_rnaseq_tpm.txt",sep="\t",header = T,check.names = F,row.names = 1)

tumor_sample<-sort(colnames(luad_tpm)[(which(substring(colnames(luad_tpm),14,15)<=10))])

filter<-which(duplicated(substring(tumor_sample,1,14)))

tumor_sample_filter<-tumor_sample[-filter]

normal_sample<-sort(colnames(luad_tpm)[(which(substring(colnames(luad_tpm),14,15)>10))])

tumor_data<-luad_tpm %>% as_tibble() %>% dplyr::select(one_of(tumor_sample_filter))

data<-data.frame(row.names=rownames(luad_fpkm),tumor_data,check.names = FALSE)

gene<-row.names(data)

#which(row.names(data)=="ENSG00000170779")

data_trans<-t(data)

#data_trans[,"ENSG00000170779"]

#cor.test(x=datat1[,1],y=datat1[,2])

options(scipen = 10)#取消科学记数法，10位以内

all<-data.frame()

for (i in 1:length(gene)){

cor<-cor.test(x=data_trans[,"ENSG00000170779"],y=data_trans[,gene[i]])

#print(cor)

result<-c("ENSG00000170779",gene[i],cor$estimate,cor$p.value)

all<-rbind(all,result)

}

colnames(all) <- c("CDCA4","ENSEMBL","Corr","P_value")

all_new<-all

all_new$Corr<-as.numeric(all_new$Corr)

all_new$P_value<-as.numeric(all_new$P_value)

symbol=bitr(all_new$ENSEMBL, 'ENSEMBL', "SYMBOL", "org.Hs.eg.db",drop=FALSE)

all_final<-left_join(all_new,symbol,by="ENSEMBL")

write.table(all_final, file="/Users/zhaoguofeng/work-Geoff/personal/Tan_Hunan_Clinical_hospital_LUAD_ceRNA/TCGA-LUAD/CDCA4_TPM_corr_gene.txt",sep="\t")

Cor_gene_list<-all_new$ENSEMBL[which((all_new$P_value<0.05) & (abs(all_new$Corr) > 0.2))]

length(Cor_gene_list)

#gene_list1<-all_new$ENSEMBL[which((all_new$P_value<0.05) & all_new$Corr> 0.2)]

#gene_list2<-all_new$ENSEMBL[which((all_new$P_value<0.05) & all_new$Corr< -0.2)]

Cor_gene_list=bitr(Cor_gene_list, 'ENSEMBL', "ENTREZID", "org.Hs.eg.db")[, "ENTREZID"]

#########################################################

#KEGG enrichment analysis #

#########################################################

ORA_KEGG <-enrichKEGG(Cor_gene_list,

keyType = "kegg",

pvalueCutoff=0.05,

qvalueCutoff=0.2,

pAdjustMethod = "BH",

minGSSize = 5,

maxGSSize = 500,

organism = "hsa",

use_internal_data=FALSE)

ORA_KEGG <-setReadable(ORA_KEGG, OrgDb = org.Hs.eg.db, keyType="ENTREZID")

write.csv(ORA_KEGG,"/Users/zhaoguofeng/work-Geoff/personal/Tan_Hunan_Clinical_hospital_LUAD_ceRNA/TCGA-LUAD/CDCA4-TPM_corgene_KEGG_enrichment.csv",row.names =F)

pdf("/Users/zhaoguofeng/work-Geoff/personal/Tan_Hunan_Clinical_hospital_LUAD_ceRNA/TCGA-LUAD/CDCA4-TPM_corgene_KEGG_enrichment.pdf",width=10,onefile=F)

print(barplot(ORA_KEGG, showCategory=30,title="Barplot of KEGG Enrichment"))

dev.off()

tiff("/Users/zhaoguofeng/work-Geoff/personal/Tan_Hunan_Clinical_hospital_LUAD_ceRNA/TCGA-LUAD/CDCA4-TPM_corgene_KEGG_enrichment.tiff")

print(barplot(ORA_KEGG, showCategory=30,title="Barplot of KEGG Enrichment"))

dev.off()

#########################################################

#GO enrichment analysis #

#########################################################

All<-c("BP","CC","MF")

for(i in 1:3){

ORA_GO<-enrichGO(gene=Cor_gene_list,

OrgDb = "org.Hs.eg.db",

keyType = "ENTREZID",

ont= All[i],

pAdjustMethod = "BH",

pvalueCutoff = 0.05,

qvalueCutoff = 0.2,

readable= TRUE)

csv_name<-paste0("/Users/zhaoguofeng/work-Geoff/personal/Tan_Hunan_Clinical_hospital_LUAD_ceRNA/TCGA-LUAD/","CDCA4-TPM_corgene_GO_enrichment_",All[i],".csv")

write.csv(ORA_GO,csv_name,row.names =F)

pdf_name1<-paste0("/Users/zhaoguofeng/work-Geoff/personal/Tan_Hunan_Clinical_hospital_LUAD_ceRNA/TCGA-LUAD/","CDCA4-TPM_corgene_GO_enrichment_",All[i],"_bar.pdf")

pdf(pdf_name1,width=10,onefile=F)

title<-paste0("Barplot of ", All[i] ," Enrichment")

print(barplot(ORA_GO, showCategory=20,title=title))

dev.off()

pdf_name2<-paste0("/Users/zhaoguofeng/work-Geoff/personal/Tan_Hunan_Clinical_hospital_LUAD_ceRNA/TCGA-LUAD/","CDCA4-TPM_corgene_GO_enrichment_",All[i],"_dot.pdf")

pdf(pdf_name2,width=10,onefile=F)

title<-paste0("Dotplot of ", All[i] ," Enrichment")

print(dotplot(ORA_GO, showCategory=20,title=title))

dev.off()

tiff_name1<-paste0("/Users/zhaoguofeng/work-Geoff/personal/Tan_Hunan_Clinical_hospital_LUAD_ceRNA/TCGA-LUAD/","CDCA4-TPM_corgene_GO_enrichment_",All[i],"_bar.tiff")

tiff(tiff_name1, width=800,height = 800)

title<-paste0("Barplot of ", All[i] ," Enrichment")

print(barplot(ORA_GO, showCategory=20,title=title))

dev.off()

tiff_name2<-paste0("/Users/zhaoguofeng/work-Geoff/personal/Tan_Hunan_Clinical_hospital_LUAD_ceRNA/TCGA-LUAD/","CDCA4-TPM_corgene_GO_enrichment_",All[i],"_dot.tiff")

tiff(tiff_name2, width=800,height = 800)

title<-paste0("Dotplot of ", All[i] ," Enrichment")

print(dotplot(ORA_GO, showCategory=20,title=title))

dev.off()

}

original_gene_list <- all_new$Corr[which(is.na(all_new$Corr)=="FALSE")]

names(original_gene_list) <- all_new$ENSEMBL[which(is.na(all_new$Corr)=="FALSE")]

gene_list = sort(original_gene_list,decreasing = TRUE)

#keytypes(org.Hs.eg.db)

#########################################################

#BP GSEA analysis #

#########################################################

ego <- gseGO(geneList = gene_list,

OrgDb = org.Hs.eg.db,

ont = "BP",

keyType ="ENSEMBL",

minGSSize = 500,

maxGSSize = 1000,

pvalueCutoff = 0.05,

eps = 0,

verbose = FALSE)

ego_trans <-setReadable(ego, OrgDb = org.Hs.eg.db, keyType="ENSEMBL")

write.csv(ego_trans,"/Users/zhaoguofeng/work-Geoff/personal/Tan_Hunan_Clinical_hospital_LUAD_ceRNA/TCGA-LUAD/CDCA4_TPM_group_gseGO_enrichment_BP.csv",row.names =F)

gseaGO_length<-length(ego[,"ID"])

for(i in 1:gseaGO_length){

lable<-paste0("NES = ",signif(ego$NES[i],3),"\npvalue = ",signif(ego$pvalue[i],3),"\nqvalue = ",signif(ego$qvalues[i],3))

grob <- grobTree(textGrob(lable, x=0.8, y=0.9, hjust=0,gp=gpar(col="red", fontsize=10)))

pdf_name=paste0("/Users/zhaoguofeng/work-Geoff/personal/Tan_Hunan_Clinical_hospital_LUAD_ceRNA/TCGA-LUAD/CDCA4-TPM_group_gseGO_goset_",i,".pdf")

pdf(pdf_name,width=10,onefile=F)

print(gseaplot2(ego,geneSetID = i,title = ego$Description[i])+annotation_custom(grob))

dev.off()

tiff_name=paste0("/Users/zhaoguofeng/work-Geoff/personal/Tan_Hunan_Clinical_hospital_LUAD_ceRNA/TCGA-LUAD/CDCA4-TPM_group_gseGO_goset_",i,".tiff")

tiff(tiff_name,width=800,height = 800)

print(gseaplot2(ego,geneSetID = i,title = ego$Description[i])+annotation_custom(grob))

dev.off()

}

pdf("/Users/zhaoguofeng/work-Geoff/personal/Tan_Hunan_Clinical_hospital_LUAD_ceRNA/TCGA-LUAD/CDCA4-TPM_group_gseGO_enrichment_BP_ridgeplot.pdf",width=10,height=15,onefile=F)

print(ridgeplot(ego))

dev.off()

tiff("/Users/zhaoguofeng/work-Geoff/personal/Tan_Hunan_Clinical_hospital_LUAD_ceRNA/TCGA-LUAD/CDCA4-TPM_group_gseGO_enrichment_BP_ridgeplot.pdf",width=800,height=800)

print(ridgeplot(ego))

dev.off()

pdf("/Users/zhaoguofeng/work-Geoff/personal/Tan_Hunan_Clinical_hospital_LUAD_ceRNA/TCGA-LUAD/CDCA4-TPM_group_gseGO_enrichment_BP_dotplot.pdf",width=10,height=15,onefile=F)

print(dotplot(ego,showCategory = 30))

dev.off()

tiff("/Users/zhaoguofeng/work-Geoff/personal/Tan_Hunan_Clinical_hospital_LUAD_ceRNA/TCGA-LUAD/CDCA4-TPM_group_gseGO_enrichment_BP_dotplot.pdf",width=800,height=800)

print(dotplot(ego,showCategory = 30))

dev.off()

resevre_all<-all_new[which(is.na(all_new$Corr)=="FALSE"),]

trans_gene<-bitr(resevre_all$ENSEMBL, 'ENSEMBL', "ENTREZID", "org.Hs.eg.db",drop=T)

keep_gene <- dplyr::distinct(trans_gene,ENTREZID,.keep_all=T)

gene_df <- data.frame(Corr=resevre_all$Corr,

ENSEMBL =resevre_all$ENSEMBL)

gene_df <- merge(gene_df,keep_gene,by='ENSEMBL')

geneList <- as.numeric(gene_df$Corr)

names(geneList) = gene_df$ENTREZID

sorted_gene_list = sort(geneList,decreasing = TRUE)

#########################################################

#KEGG GSEA analysis #

#########################################################

ekegg <- gseKEGG(geneList = sorted_gene_list,

organism = 'hsa',

minGSSize = 10,

maxGSSize = 500,

pvalueCutoff = 0.05,

eps = 0,

verbose = FALSE)

ekegg_trans <-setReadable(ekegg, OrgDb = org.Hs.eg.db, keyType="ENTREZID")

gsekegg_length<-length(ekegg[,"ID"])

write.csv(ekegg_trans,"/Users/zhaoguofeng/work-Geoff/personal/Tan_Hunan_Clinical_hospital_LUAD_ceRNA/TCGA-LUAD/CDCA4-TPM_group_gseKEGG_enrichment.csv",row.names =F)

for(i in 1:gsekegg_length){

lable<-paste0("NES = ",signif(ekegg$NES[i],3),"\npvalue = ",signif(ekegg$pvalue[i],3),"\nqvalue = ",signif(ekegg$qvalues[i],3))

grob <- grobTree(textGrob(lable, x=0.8, y=0.9, hjust=0,gp=gpar(col="red", fontsize=10)))

pdf_name=paste0("/Users/zhaoguofeng/work-Geoff/personal/Tan_Hunan_Clinical_hospital_LUAD_ceRNA/TCGA-LUAD/CDCA4-TPM_group_gseKEGG_keggset_",i,".pdf")

pdf(pdf_name,width=10,onefile=F)

P<-gseaplot2(ekegg,geneSetID = i,title = ekegg$Description[i])+annotation_custom(grob)

print(P)

dev.off()

tiff_name=paste0("/Users/zhaoguofeng/work-Geoff/personal/Tan_Hunan_Clinical_hospital_LUAD_ceRNA/TCGA-LUAD/CDCA4-TPM_group_gseKEGG_keggset_",i,".tiff")

pdf(tiff_name)

P<-gseaplot2(ekegg,geneSetID = i,title = ekegg$Description[i])+annotation_custom(grob)

print(P)

dev.off()

}

pdf("/Users/zhaoguofeng/work-Geoff/personal/Tan_Hunan_Clinical_hospital_LUAD_ceRNA/TCGA-LUAD/CDCA4-TPM_group_gseKEGG_ridgeplot.pdf",width=10,onefile=F)

print(ridgeplot(ekegg))

dev.off()

tiff("/Users/zhaoguofeng/work-Geoff/personal/Tan_Hunan_Clinical_hospital_LUAD_ceRNA/TCGA-LUAD/CDCA4-TPM_group_gseKEGG_ridgeplot.tiff")

print(ridgeplot(ekegg))

dev.off()

pdf("/Users/zhaoguofeng/work-Geoff/personal/Tan_Hunan_Clinical_hospital_LUAD_ceRNA/TCGA-LUAD/CDCA4-TPM_group_gseKEGG_dotplot.pdf",width=10,onefile=F)

print(dotplot(ekegg,showCategory = 30))

dev.off()

tiff("/Users/zhaoguofeng/work-Geoff/personal/Tan_Hunan_Clinical_hospital_LUAD_ceRNA/TCGA-LUAD/CDCA4-TPM_group_gseKEGG_dotplot.tiff")

print(dotplot(ekegg,showCategory = 30))

dev.off()

epathway<-gsePathway(sorted_gene_list,

pvalueCutoff = 0.05,

pAdjustMethod = "BH",

eps = 0,

verbose = FALSE)

epathway_trans <-setReadable(epathway, OrgDb = org.Hs.eg.db, keyType="ENTREZID")

write.csv(ekegg_trans,"/Users/zhaoguofeng/work-Geoff/personal/Tan_Hunan_Clinical_hospital_LUAD_ceRNA/TCGA-LUAD/CDCA4-TPM_group_gseReactome_enrichment.csv",row.names =F)

pdf("/Users/zhaoguofeng/work-Geoff/personal/Tan_Hunan_Clinical_hospital_LUAD_ceRNA/TCGA-LUAD/CDCA4-TPM_group_gseReactome_cnetplot.pdf",width=10,onefile=F)

cnetplot(epathway_trans, categorySize="pvalue", foldChange=sorted_gene_list,showCategory=5,colorEdge = TRUE)

dev.off()

tiff("/Users/zhaoguofeng/work-Geoff/personal/Tan_Hunan_Clinical_hospital_LUAD_ceRNA/TCGA-LUAD/CDCA4-TPM_group_gseReactome_cnetplot.tiff")

cnetplot(epathway_trans, categorySize="pvalue", foldChange=sorted_gene_list,showCategory=5,colorEdge = TRUE)

dev.off()

epathway2 <- pairwise_termsim(epathway)

pdf("/Users/zhaoguofeng/work-Geoff/personal/Tan_Hunan_Clinical_hospital_LUAD_ceRNA/TCGA-LUAD/CDCA4-TPM_group_gseReactome_pairwise_termsim.pdf",width=10,onefile=F)

print(emapplot(epathway2,layout = "kk"))

dev.off()

epathway2 <- pairwise_termsim(epathway)

tiff("/Users/zhaoguofeng/work-Geoff/personal/Tan_Hunan_Clinical_hospital_LUAD_ceRNA/TCGA-LUAD/CDCA4-TPM_group_gseReactome_pairwise_termsim.tiff")

print(emapplot(epathway2,layout = "kk"))

dev.off()

#infiltration analysis of cibersort

luad_tpm<-read.table(file="/Users/zhaoguofeng/work-Geoff/personal/Tan_Hunan_Clinical_hospital_LUAD_ceRNA/TCGA-LUAD/LUAD_rnaseq_tpm.txt",sep="\t",header = T,check.names = F,row.names = 1)

tumor_sample<-sort(colnames(luad_tpm)[(which(substring(colnames(luad_tpm),14,15)<=10))])

filter<-which(duplicated(substring(tumor_sample,1,14)))

tumor_sample_filter<-substring(tumor_sample[-filter],1,15)

tumor_data_tmp<-luad_tpm %>% as_tibble() %>% dplyr::select(one_of(tumor_sample[-filter]))

tumor_data_final<-data.frame(row.names=rownames(luad_tpm),tumor_data_tmp,check.names = FALSE)

colnames(tumor_data_final)<-substring(colnames(tumor_data_final),1,15)

luad_ENSG00000170779<-as.data.table(t(tumor_data_final[which(row.names(tumor_data_final)=="ENSG00000170779"),]),keep.rownames="sample")

#input infiltration result of timer2

infiltration<-read.table(file="/Users/zhaoguofeng/work-Geoff/personal/Tan_Hunan_Clinical_hospital_LUAD_ceRNA/TCGA-LUAD/infiltration_estimation_for_tcga.csv",sep=",",header=TRUE,row.names = 1,check.names = F)

cibersort_tcga<-infiltration %>% as_tibble() %>% dplyr::select(7:28)

final_cibersort_tcga<-data.frame(row.names=rownames(infiltration),cibersort_tcga,check.names = FALSE)

tmp<-as.data.frame(t(final_cibersort_tcga))

cibersort_luad<-tmp %>% as_tibble() %>% dplyr::select(one_of(tumor_sample_filter))

final_cibersort_luad<-as.data.table(t(data.frame(row.names=rownames(tmp),cibersort_luad,check.names = FALSE)),keep.rownames="sample")

all_final<-as.data.frame(left_join(final_cibersort_luad,luad_ENSG00000170779,by="sample"))

colnames(all_final)<-gsub(pattern = "\\s+", replacement = "_", colnames(all_final),perl = TRUE)

cell_type=colnames(all_final)

length<-length(colnames(all_final))-1

cor_result<-data.frame()

for (i in 2:length){

print (i)

cor<-cor.test(x=all_final[,"ENSG00000170779"],y=all_final[,cell_type[i]])

print(cor)

result<-c("ENSG00000170779",cell_type[i],cor$estimate,cor$p.value)

cor_result<-rbind(cor_result,result)

}

colnames(cor_result) <- c("ENSG00000170779","cell_type","Corr","P_value")

cor_result$Corr<-as.numeric(cor_result$Corr)

cor_result$P_value<-as.numeric(cor_result$P_value)

cor_result$logP_value<-as.numeric(-log10(cor_result$P_value))

cor_result$abs_Corr<-as.numeric((abs(cor_result$Corr)))

write.csv(cor_result,"/Users/zhaoguofeng/work-Geoff/personal/Tan_Hunan_Clinical_hospital_LUAD_ceRNA/TCGA-LUAD/CDCA4-TPM_cor_infiltration_result.csv",row.names =F)

cor_result[which((cor_result$P_value< 0.05) & (abs(cor_result$Corr)>0.1)),]

P<-ggdotchart(cor_result,x="cell_type",y="Corr",add = "segments", dot.size = "abs_Corr",color="logP_value")+gradient_color(c("blue","red"))

ggpar(P,legend.title = "-log10(P_value)")

dotchart=paste0("/Users/zhaoguofeng/work-Geoff/personal/Tan_Hunan_Clinical_hospital_LUAD_ceRNA/TCGA-LUAD/CDCA4-TPM_Correlation_Cibersort-dot.pdf")

pdf(dotchart,width=10,onefile=F)

P<-ggdotchart(cor_result,x="cell_type",y="Corr",add = "segments", dot.size = "abs_Corr",color="logP_value")+gradient_color(c("blue","red"))

print(ggpar(P,legend.title="-log10(P_value)"))

dev.off()

dotchart1=paste0("/Users/zhaoguofeng/work-Geoff/personal/Tan_Hunan_Clinical_hospital_LUAD_ceRNA/TCGA-LUAD/CDCA4-TPM_Correlation_Cibersort-dot.tiff")

tiff(dotchart1)

P<-ggdotchart(cor_result,x="cell_type",y="Corr",add = "segments", dot.size = "abs_Corr",color="logP_value")+gradient_color(c("blue","red"))

print(ggpar(P,legend.title="-log10(P_value)"))

dev.off()

colnames(all_final)<-gsub(pattern = "\\+|\\(|\\)", replacement = "", colnames(all_final),perl = TRUE)

cell_type=colnames(all_final)

for (i in 2:length){

name=paste0("/Users/zhaoguofeng/work-Geoff/personal/Tan_Hunan_Clinical_hospital_LUAD_ceRNA/TCGA-LUAD/","CDCA4-TPM_",cell_type[i],".pdf")

pdf(name,onefile=F)

print(ggscatter(all_final,x="ENSG00000170779",y=cell_type[i],cor.coef = TRUE,cor.method = "pearson",add = "reg.line",conf.int = TRUE,add.params = list(color = "red", fill = "lightgray")))

dev.off()

name1=paste0("/Users/zhaoguofeng/work-Geoff/personal/Tan_Hunan_Clinical_hospital_LUAD_ceRNA/TCGA-LUAD/","CDCA4-TPM_",cell_type[i],".tiff")

tiff(name1)

print(ggscatter(all_final,x="ENSG00000170779",y=cell_type[i],cor.coef = TRUE,cor.method = "pearson",add = "reg.line",conf.int = TRUE,add.params = list(color = "red", fill = "lightgray")))

dev.off()

}
