# Supplementary figures and images for: CDCA4 as a novel molecular biomarker of poor prognosis in patients with lung adenocarcinoma
Source: Front Oncol. 2022 Sep 15;12:865756. doi: 10.3389/fonc.2022.865756 (PMC9520321; doi:10.3389/fonc.2022.865756)

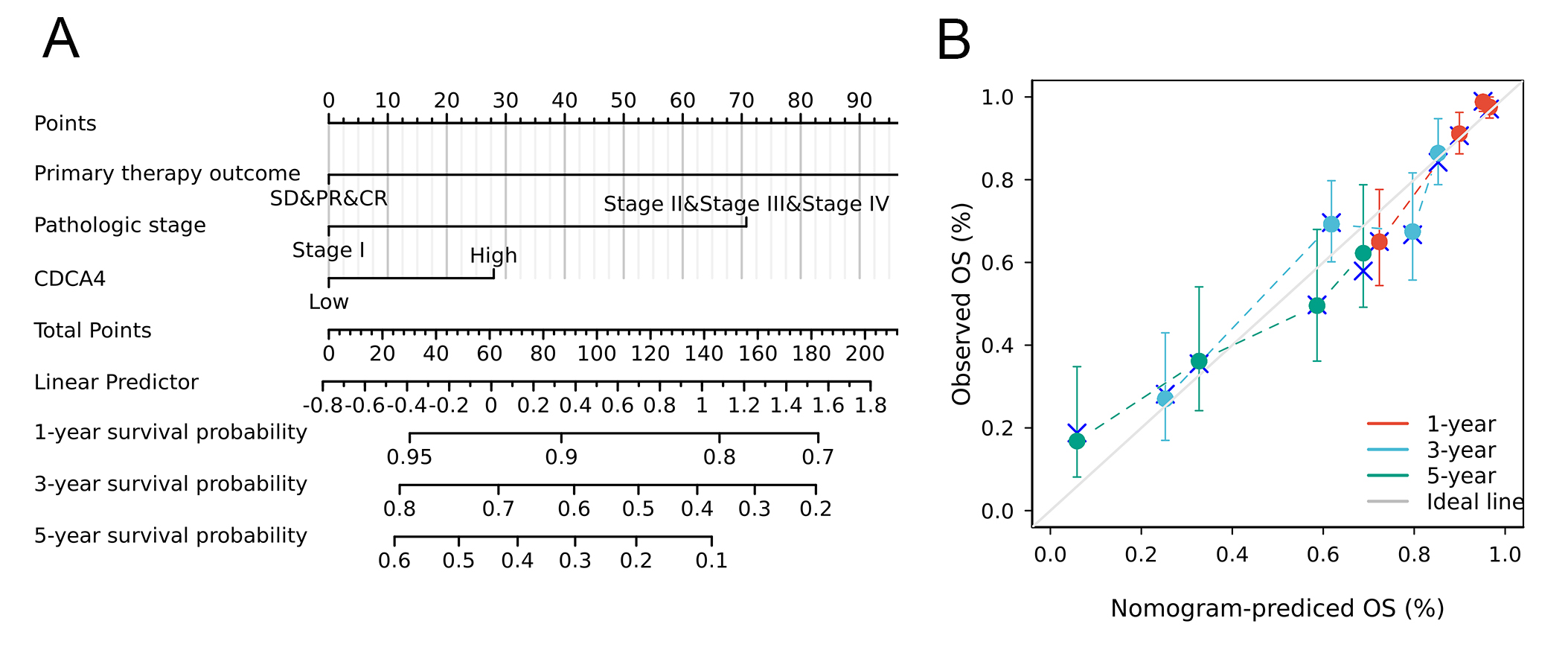

Supplement: Supplementary file 8 [file Image_1.jpeg]

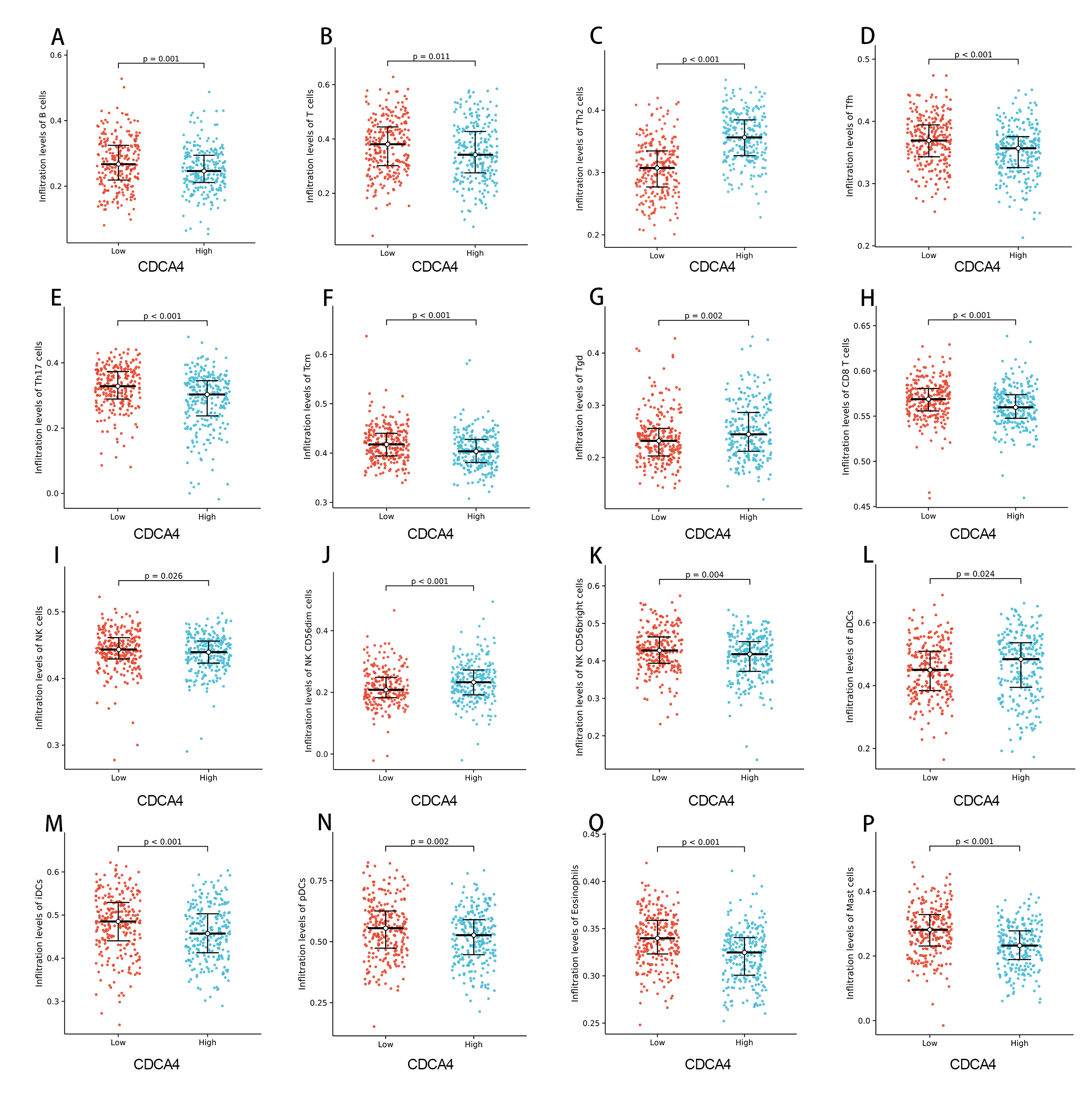

Supplement: Supplementary file 9 [file Image_2.jpeg]
