# Supplementary material for: CDCA4 as a novel molecular biomarker of poor prognosis in patients with lung adenocarcinoma
Source: Front Oncol. 2022 Sep 15;12:865756. doi: 10.3389/fonc.2022.865756 (PMC9520321; doi:10.3389/fonc.2022.865756)
Supplement: Supplementary file 10 [file Table_1.docx]

**Table S1. The details of subjects for RT-qPCR and WB**

| **No.** | **Gender** | **Age** | **T** | **N** | **M** | **Stage** | **Neoadjuvant**  **therapy** | **IASLC grade** |
| --- | --- | --- | --- | --- | --- | --- | --- | --- |
| 1 | Male | 69 | T1a | 0 | 0 | IA1 | No | 3 |
| 2 | Male | 70 | T2a | 2 | 0 | IIIA | No | 2 |
| 3 | Female | 56 | T1b | 0 | 0 | IA2 | No | 1 |
| 4 | Female | 45 | T1a | 0 | 0 | IA1 | No | 2 |
| 5 | Male | 54 | T2a | 0 | 0 | IB | No | 3 |
| 6 | Female | 63 | T2b | 1 | 0 | IIB | No | 1 |
| 7 | Female | 62 | T2a | 0 | 0 | IB | No | 3 |
| 8 | Male | 60 | T2a | 0 | 0 | IB | No | 2 |
| 9 | Male | 55 | T1c | 0 | M1a | IVA | No | 3 |
| 10 | Male | 58 | T2a | 0 | 0 | IB | No | 1 |

T, T-stage; N, N-stage; M, M stage; stage, the American Joint Committee on Cancer (AJCC) 8th edition staging manual for lung cancer; IASLC, International Association for the Study of Lung Cancer.
