# Supplementary material for: CDCA4 as a novel molecular biomarker of poor prognosis in patients with lung adenocarcinoma
Source: Front Oncol. 2022 Sep 15;12:865756. doi: 10.3389/fonc.2022.865756 (PMC9520321; doi:10.3389/fonc.2022.865756)
Supplement: Supplementary file 12 [file Table_3.docx]

Table S3 Univariate and multivariate analysis of the relationship between overall survival and clinicopathologic characteristics in patients with TCGA.

| **Characteristics** | **Total number(N)** | **HR (95% CI)** | ***P* value** |
| --- | --- | --- | --- |
| A |  |  |  |
| Age (>65 vs. <=65) | 494 | 1.228(0.915-1.649) | 0.171 |
| Gender (Female vs. Male) | 504 | 0.943(0.705-1.262) | 0.694 |
| Smoker (Yes vs. No) | 490 | 0.887(0.587-1.339) | 0.568 |
| number pack years smoked (>=40 vs. <40) | 345 | 1.038(0.723-1.490) | 0.84 |
| T stage (T2-T4 vs. T1) | 501 | 1.668(1.184-2.349) | 0.003 |
| N stage (N1-3 vs. N0) | 492 | 2.606(1.939-3.503) | <0.001 |
| M stage (M1 vs. M0) | 360 | 2.111(1.232-3.616) | 0.007 |
| Pathologic stage (Stage II-IV vs. Stage I) | 496 | 2.975(2.188-4.045) | <0.001 |
| Primary therapy outcome (PD vs. SD-CR) | 419 | 3.978(2.785-5.682) | <0.001 |
| TP53 status (Mut vs. WT) | 499 | 1.254(0.936-1.680) | 0.13 |
| CDCA4 (High vs. Low) | 504 | 1.519(1.131-2.040) | 0.006 |
| B |  |  |  |
| Pathologic stage (Stage II-IV vs. Stage I) | 496 | 2.462(1.731-3.501) | <0.001 |
| Primary therapy outcome (PD vs. SD-CR) | 419 | 3.570(2.472-5.155) | <0.001 |
| CDCA4 (High vs. Low) | 504 | 1.427(1.017-2.003) | 0.04 |
